# Protective Power of Vaccination

**Published:** 1861-03

**Authors:** H. Noble

**Affiliations:** Heyworth


### PROTECTIVE POWER OF VACCINATION.

BY H. NOBLE, M. D.

HEYWORTH, March 6, 1861.

MR. EDITOR:—I desire to offer to the profession through the *Journal* some observations made several years ago which illustrated the protective power of Vaccina.

The result of the cases treated by me at that time and the effect produced on those exposed to infection, led me to adopt some additions to the theory of protection which I have not seen taught by the standards or in monograph.

I will in the first place state that I believe Vaccina affords perfect protection from Variola; next, that protection once obtained by Vaccina continues undiminished through life. Third—That perfect protection can be obtained by repeated vaccinations, and that the proper time to renew the operation is as soon as the vaccine disease has run its course. No evidence of the efficacy of the vaccine disease is satisfactory until it is ascertained by trial that the vaccine virus has no more effect. Fourth—The protection does not depend on the size, form or character of pustule, or indeed on the existence of the pustule at all, but on the constitutional disease.

I know that this last position is very different from the opinion of many of my confreres who have often been known to gravely inspect the cicatrix left by the pustule many years before to ascertain if the disease had been '*genuine*.'

But let me give a few of the cases and see if they sustain the positions taken.

In the fall of 1849 a pauper woman and her infant were admitted into a family of thirteen persons. The woman was infected with small pox, and on the fifth day after breaking out I saw her for the first time. It was a case of confluent small pox, the centers were depressed and filling with pus.

The family consisted of the father, mother, a widow lady, and ten children. The father had had vaccine disease twenty-five years before; the mother and widow each thirty years before, and three of the children had been vaccinated one year previously, but one of them did not succeed. This left eight children in the family and the pauper's infant exposed to small pox without protection.

In addition to the family mentioned, there was a family visiting at the sick house consisting of father and mother and six children. Of this last family the father and mother were protected by vaccina, the six children unprotected.

On the morning of the fifth day after I first saw them I procured vaccine virus and vaccinated all that had been exposed.

None of those protected by vaccina had small pox. The father, who had had vaccina twenty-five years previously, had the initiatory fever, which was followed by two or three pox, which aborted, and he had no secondary fever. One of the children that had vaccina one year previously, had varioloid, with secondary fever. None of the other protected members of the family were sick.

The eight unprotected children of the family were well when vaccinated, and the vaccina disease progressed favorably from five to seven days—that is, the first was attacked with small pox on the fifth, and the last on the seventh day after vaccination.

The vaccina disease progressed during that time favorably, and, as far as I could perceive, without modification from the incubating variola, but from the invasion of the variola fever the vaccina pustules remained stationary until the subsidence of the secondary fever, when the partly formed vaccina pustules proceeded to maturity, each case having a fully developed vaccina pustule.

In one case a small pox pustule impinged on a partly formed vaccina pustule, which vaccina pustule matured apparently as perfectly after the small pox declined as any case of uncomplicated vaccina.

The family of six children mentioned as visitors were vaccinated on the fifth day after exposure, and they all had vaccina and no small pox or varioloid.

In another family a case of small pox occurred, in which was a child unprotected. I vaccinated the child on the third day after the eruption of small pox, and the child stayed in the room with the sick man all the time. The child had vaccina unmodified, the protection being as if it had had vaccina a year before. In this last mentioned family the man had had vaccina thirty years previously, and the woman fifteen, and neither of them were sick.

These cases, I think, show conclusively that the protection of vaccina does not run out every seven, or fourteen, or twenty-one years, or at any septennial period. Three of the cases showing the protection to be complete for thirty years, one complete for fifteen years, one partial for twenty-five years, and one partial for one year. In addition to these, my own protection was vaccina, which was of 32 years standing.

None of these cases of complete or partial protection had been revaccinated, except my own. Now these are not many cases to base an opinion upon, but they correspond with an observation of twelve or fifteen years, during which time I

have seen many cases that were protected by repeated vaccinations exposed to small-pox, not one of which were disturbed by the disease.

For the last ten years, I have been somewhat careful to observe the proportion of cases in which a second vaccination takes effect, and my judgment is that about one-third of the second vaccinations produce pustules more or less perfect, the constitutional disturbance being always less than in the first cases. Not more than one in fifty of my third vaccinations produce any effect.

Suppose that as long as there is any susceptibility to variola in the system, vaccination acts on that susceptibility and destroys it, (which I believe,) it is evident that revaccination should be resorted te until it would have no more effect, which would be the evidence that the susceptibility was destroyed.

This is the point that I wish especially to present to the profession, that full protection can be obtained by vaccina, and when that protection is secured, it can be certainly known by the test of revaccination.

Assuming that the foregoing is true, it follows of course that the physician, when consulted as to the propriety and efficacy of vaccination, should give a more decided opinion in its favor than is commonly done. The certainty of full protection should be represented to be fully equal to having had small-pox itself, and not, as is often the case, represented as a good sort of precaution which will, when put to the test, disappoint you.

It is the duty of the physician to give such an opinion to the public as will establish perfect confidence in the remedy, so that in seeking it they will not have to "hope against reason," as many of them express it, and when that confidence is established in the mind of the public, vaccination will not be neglected as it has been, but will be sought and demanded by the people with the earnestness that its importance justifies.

